# Initial Genome-Wide Case–Control Study for Genetic Background of Retinal Dysplasia in Czechoslovakian Wolfdog

**DOI:** 10.3390/vetsci12020171

**Published:** 2025-02-14

**Authors:** Michal Gábor, Juraj Candrák, Martina Miluchová, Pavol Zubrický, Agnieszka Balická, Alexandra Trbolová

**Affiliations:** 1 Faculty of Agrobiology and Food Resources, Institute of Nutrition and Genomics, Slovak University of Agriculture in Nitra, Tr. A. Hlinku 2, 949 76 Nitra, Slovakia; michal.gabor@uniag.sk (M.G.); juraj.candrak@uniag.sk (J.C.); 2 Small Animal Clinic, University Veterinary Hospital, University of Veterinary Medicine and Pharmacy in Košice, Komenského 73, 041 81 Košice, Slovakia; pavol.zubricky@gmail.com (P.Z.); agnieszka.a.balicka@gmail.com (A.B.); alexandra.trbolova@uvlf.sk (A.T.)

**Keywords:** retinal dysplasia, Czechoslovakian wolfdog, GWAS analysis, gene *CYP27A1*

## Abstract

Retinal dysplasia is a genetically heterogeneous ocular disorder in dogs, characterized by abnormal retinal development, resulting in a range of visual impairments from mild to complete blindness. The objective of this study was to investigate the prevalence and genetic basis of retinal dysplasia in the Czechoslovakian Wolfdog breed. An ophthalmic examination was conducted on a cohort of 117 Czechoslovakian Wolfdogs, which revealed a prevalence of multifocal retinal dysplasia of 5.13%. A genome-wide case–control association study was conducted on a subset of 36 adult dogs to explore the underlying genetic architecture of multifocal retinal dysplasia in this breed. The GWAS identified a suggestive association with a locus on canine chromosome CFA37. The strongest association signal for SNP marker BICF2G630130992 (*p* = 1.29 × 10^−6^) was identified in the first intron of the *CYP27A1* gene, which encodes a cytochrome P450 enzyme involved in vitamin D metabolism and potentially retinal function. The region of CFA37 contains several other genes that have been previously implicated in ocular development and disease. Further studies utilizing next-generation sequencing and functional analyses are required to validate these findings, identify the causative variants, and fully elucidate the genetic architecture of retinal dysplasia in this breed.

## Simple Summary

Retinal dysplasia is a complex canine eye disease that is determined by a combination of genetic and non-genetic risk factors. The genetic basis of retinal dysplasia in specific canine breeds remains incompletely understood. While the Czechoslovakian Wolfdog is generally known for being free of significant eye problems, our research has shown that multifocal retinal dysplasia can occur in this breed. A clinical study of 117 Czechoslovakian Wolfdogs found six samples with symptoms of multifocal retinal dysplasia. A genome-wide case–control association study of 36 adult dogs identified association on chromosome CFA37, specifically in the first intron of the *CYP27A1* gene. The region of this gene is very interesting because other genes linked to ocular diseases in animal models are localized in this locus. It suggests the possibility of multiple genes contributing to the multiple retinal dysplasia phenotype in Czechoslovakian Wolfdogs.

## 1. Introduction

Retinal dysplasia (RD) is a condition in which there is a defect in the proliferation and differentiation of the developing retina [1]. It is a heterogeneous group of conditions that manifests in varying degrees, from those that do not affect vision to those that lead to blindness [2]. RD has a non-progressive character and can occur in three forms: (multi)focal, geographic, and total. The focal/multifocal form is characterised by a wide range of ophthalmoscopically visible abnormalities ranging from retinal folds appearing as streaks, dots, and/or worm-like lesions, single or multiple, and localised in tapetal or non-tapetal fundus [3]. When this form of retinal dysplasia is observed in puppies, it may resolve partially or completely by adulthood. The characteristic lesion of the geographic form of RD is a most often horseshoe-like shaped area of both retinal thinning and elevation representing retinal detachment and retinal disorganisation in the central tapetal fundus [4]. Total retinal dysplasia is described as a severe retinal disorganisation associated with the separation of the neurosensory retina from the retinal pigment epithelium [5].

It has been observed to occur as a distinct manifestation and as part of a severe inherited syndromic disorder known as oculoskeletal dysplasia [3]. Two types of retinal dysplasia associated with the occurrence of skeletal defects have been described: dwarfism with retinal dysplasia type 1 (DRD1) in the Labrador retriever, the Nordic Inuit dog, and the Australian labradoodle, and dwarfism with retinal dysplasia type 2 (DRD2) in the Samoyed. In the above types, causal mutations were found in the *COL9A2* gene in the Samoyed breed (deletion of 1267 bp in the 5’ UTR region) and the *COL9A3* gene in the Labrador retriever (insertion of 1 bp in exon 1) and Northern Inuit dog (premature termination codon) breeds [6,7]. In contrast to the type of retinal dysplasia reported above, the occurrence of retinal dysplasia without skeletal defects has been reported in several breeds of dogs. Based on ACVO Genetics Committee data evaluated over the periods 1993–2018 and 2019–2023, the highest prevalence of retinal dysplasia in the form of folds was observed in Berger Picard (17.4% and 6.5%), Cocker Spaniel (11, 5% and 3.6%) Field Spaniel (9.8% and 5%), Sussex Spaniel (9.2% and 7.3%), Black and Tan Coonhound (8.7% and 11.3%), Mastiff (7.2% and 5.7%), Collie (6.9% and 7.3%), Cavalier King Charles Spaniel (6.7% and 3.2%), or Clumber Spaniel (6.4% and 2.3%). In the case of the Czechoslovakian Wolfdog breed, only one retinal dysplasia has been reported out of 30 dogs examined between 1993–2018 (2.9%). No case has been detected in the timeframe 2019–2023. For the German Shepherd, which was used as the basis for the development of the Czechoslovak Wolfdog breed, the prevalence of retinal dysplasia was found to be 1.8% (1993–2018) and 1.3% (2019–2023) [5].

However, no causal mutations associated with the manifestation of retinal dysplasia without skeletal defects in any breed have been described so far. The occurrence of a given type of retinal dysplasia in different breeds provides a basis for the occurrence of different mutations in genes involved in the development of the ocular apparatus. The use of sophisticated molecular genetic methods can greatly accelerate the identification of genetic markers and possible causal mutations associated with the manifestation of retinal dysplasia for a particular breed of dog.

A genome-wide association study (GWAS) represents a convergence of genomic technologies, bioinformatics, and biostatistics. It employs a comprehensive scan of an individual’s entire genome to identify genetic variants associated with phenotypic expressions [8]. The fundamental objective of GWAS analysis is to undertake a comparative analysis of extensive genetic datasets between individuals exhibiting disparate phenotypic characteristics. These analyses can facilitate the elucidation of the association between specific genetic variants and the aetiology of diseases [9] or behavioural patterns in canines [10]. Furthermore, they can elucidate the influence of these variants on the biological processes occurring within the organism. Additionally, they can provide invaluable insights that inform the development of novel diagnostic and therapeutic approaches.

The objective of this pilot study was to utilise a genome-wide case–control association study to identify a potential region of interest in the genome of the Czechoslovakian Wolfdog that may be associated with a genetic predisposition for retinal dysplasia.

## 2. Materials and Methods

### 2.1. Study Samples

A comprehensive eye examination was conducted on 117 Czechoslovakian wolf dogs, comprising both sexes. Of these, 82 individuals were aged between one and thirteen years, while 35 puppies from five different litters were aged between six and seven weeks. All canines had pedigree documentation and originated from a multitude of kennels. The ophthalmological examination comprised behavioural and neuro-ophthalmological testing, measurement of intraocular pressure (Tonovet; Icare Finland, Vantaa, Finland) and tear production, and assessment of the anterior segment, iridocorneal angle, and posterior segment. The fundus was examined using mydriatics (1% Unitropic, Unimed, SK, Bratislava, Slovakia), a Heine direct ophthalmoscope (Germany), a monocular indirect ophthalmoscope (Pan Opticophthalmoscope, Welch Allyn, Skaneateles, NY, USA), and an indirect ophthalmoscope (Heine Omega 600, Optotechnik GmbH, Gilching, Germany), with a double aspheric lens (Volk 20D, Volk, Mentor, OH, USA). Individuals exhibiting ocular findings in puppyhood were re-examined at six months of age to assess the development of ocular abnormalities. Each identified ocular finding was documented using an Aurora fundus camera (Optomed, Oulu, North Ostrobothnia, Finland). In subjects with established retinal dysplasia, the recording of ocular findings was subjected to quantitative analysis using ImageJ software version 1.54k (National Institutes of Health, Bethesda, MD, USA), which enabled the mapping of the number of lesions and the percentage of retinal involvement. For the genome-wide association (GWAS), we defined retinal dysplasia as dogs with the focal/multifocal form.

### 2.2. Genome-Wide SNP Genotyping and Analyses

Genomic analysis was performed on a reference group of 36 individuals (6 cases and 30 controls) selected from the base set of 117 Czechoslovakian Wolfdogs. Biological material for molecular analyses was whole blood collected from individuals during the comprehensive examination. Genomic DNA was extracted from blood using the standard protocol of the commercial QIAamp DNA Mini Kit (Qiagen, Hilden, Germany) and normalised to a concentration of 50 ng/µl. Genome-wide genotyping was done using the high-density canine SNP chip Illumina CanineHD 230K BeadChip (Illumina, San Diego, CA, USA) via Neogen Europe Ltd. (Scotland, UK). The initial genotyping data was evaluated using the PLINK 1.9 software [11]. A total of 220,853 single-nucleotide polymorphism (SNP) markers were subjected to analysis and were common to all 36 individuals. The obtained raw data set was treated by selecting autosomal SNP markers localised on 38 chromosomes, with consideration given to the frequency of the minor allele frequency (MAF < 0.05) and the quality of the induced base (<0.90). The resulting set after qualitative correction consisted of 108,070 SNP markers and was achieved for all 36 individuals of the reference group. Single-nucleotide polymorphisms (SNPs) located on the X chromosome were excluded due to allelic imbalance.

For a more objective assessment of the phenotypic categorization of retinal dysplasia in positive samples, a transformation to a quantitative trait was performed by regression. The regression model included a linear and quadratic fit of the relative value of the damage to the eye and a linear and quadratic fit of the number of lesions. The Restricted Maximum Likelihood (REML) method was used to estimate the variance component by incorporating SNP information obtained after qualitative correction. Subsequently, the BLUPF90 software package [12,13] with genomic capabilities was used, incorporating the use of the preGSf90 genomic preprocessor to combine genomic and pedigree relationships, genomic quality control, removal of SNPs from chromosomes, and deposition of pure SNPs, continuing with the use of the BLUPF96 genomic module to generate an animal solution with genomic information and the postGSf90 genomic postprocessor to extract SNP solutions after genomic evaluations. A conservative Bonferroni-corrected genome-wide significance threshold of 4.63 × 10^−7^ was calculated, correcting for the number of all autosomal variants remaining after quality control (n = 108,070). A suggestive significance threshold was set at the standard *p*-value of 1.0 × 10^−5^. The Manhattan plot was generated using R program code [14]. The same linear animal model (one random effect of animals with pedigree and SNP information) was used for all analyses and estimations.

## 3. Results

### 3.1. Clinical Examination

All dogs were privately owned pet dogs. Dogs were vaccinated and dewormed following the schedules. Food was given to the dogs in different quality and quantity following owners’ preferences. The water was given ad libitum. Body condition score (BCS) was 4–5/9 in all cases. All animals were healthy dogs based on physical examination with no history of vision impairment or ocular abnormality.

**Figure 1:**
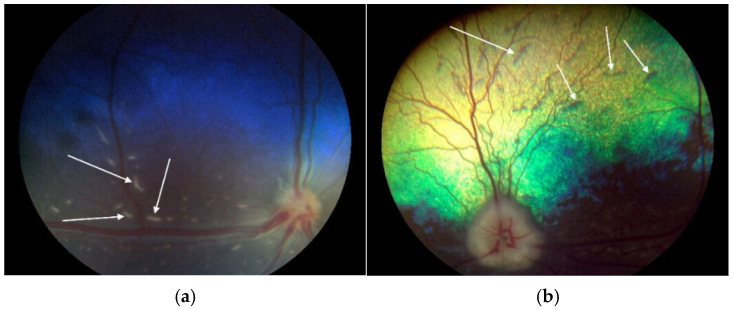
Clinical eye examination record using Aurora fundus camera (Optomed, Finland). (**a**) Retinal fold in a puppy, (**b**) multifocal form of retinal dysplasia in an adult dog. Arrows highlight clinical manifestations of each form (source: Pavol Zubrický).

In the course of our study, we examined 117 Czechoslovakian Wolfdogs and identified nine individuals (7.69%) with retinal dysplasia. Three individuals (2.56%) exhibited retinal folds and were aged between six and seven weeks at the time of examination. At the follow-up examination at 6 months, their retinas were normal; suggesting that retinal folds may resolve with time [15]. In adult Czechoslovakian Wolfdogs, multifocal retinal dysplasia was identified in six cases (5.13%), (Figure 1).

In the quantitative assessment of ocular findings, the average proportion of retinal damage in pups was 1.06%. In adults with findings of the multifocal form, the proportion of damage was 3.59%. Conversely, the low proportion of retinal damage in the form of retinal folds observed in pups indicates that retinal maturation results in the disappearance of the defects. For this reason, pups were not included in the genomic analysis.

### 3.2. Genome-Wide Case–Control Association Study

The data from the clinical measurements of adult dogs that tested positive for the multifocal form of retinal dysplasia were categorised using a regression model (Table 1) to quantitative trait and subsequently used for the genome-wide case–control association study. The mean proportion of retinal dysplasia in adult dogs with multifocal form was 2.33%.

**Figure 2:**
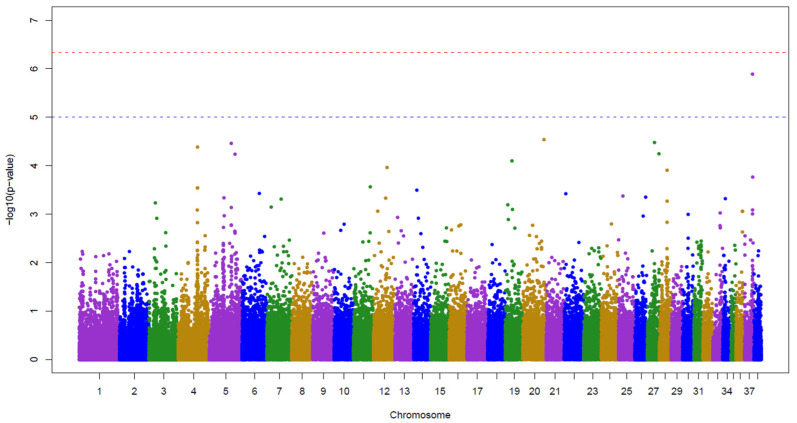
SNP associations with retinal dysplasia in Czechoslovakian Wolfdog.

The initial association analysis of retinal dysplasia in Czechoslovakian Wolfdog did not reveal significant associations after Bonferroni correction (Figure 2), but we observed a suggestive association on CFA37. The SNP marker BICF2G630130992 (*p* = 1.29 × 10^−6^) is positioned at CFA37:25,383,468 (CanFam3.1), within the first intron of the *CYP27A1* gene.

The Manhattan plot shows -log10 *p* values for SNP associations. The horizontal blue line represents a suggestive significance threshold *p*  =  1 × 10^−5^ and the horizontal red line represents a conservative Bonferroni-corrected genome-wide significance threshold *p*  =  4.63 × 10^−7^ for 108,070 SNP markers. SNPs are colour-coded by chromosome. The BICF2G630130992 (*p* = 1.29 × 10^−6^) is localised above the horizontal blue line.

**Figure 3:**
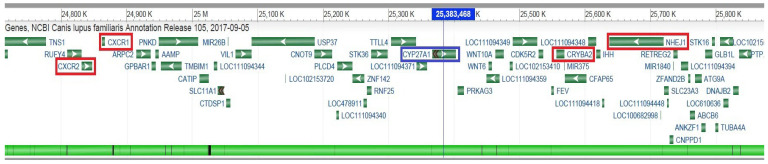
Schematic representation of the genomic region on canine chromosome CFA37 associated with retinal dysplasia in Czechoslovakian Wolfdog using genome-wide association analysis (GWAS).

Other genes whose increased expression or mutations have resulted in clinical signs of retinal damage (*CXCR1*, *CXCR2*) or cataracts in humans and animal models (*CRYBA2*) or have been directly associated with CEA disease in breeds related to Collies (*NHEJ1*) are located within 1 Mb of the *CYP27A1* gene from upstream and downstream (Figure 3).

The overview of the entire region demonstrates the position of BICF2G630130992 in the *CYP27A1* gene which was identified as a tentative association with retinal dysplasia in the population of 36 samples of the Czechoslovakian Wolfdog. The *CYP27A1* gene position is illustrated with a blue frame and the BICF2G630130992 is highlighted by the blue axis with the position 25,383,468. The other genes such as *CXRC1*, *CXRC2*, *CRYBA2*, and *NHEJ1*, associated with the development of the eye, localised up to ∼ 1 Mb downstream and upstream of the *CYP27A1* gene are shown by the red frame. The graphics of the genomic area are from the ENSEMBL website based on the CanFam3.1 assembly (https://www.ncbi.nlm.nih.gov/gdv/browser/genome/?id=GCF_000002285.3) (accessed on 4 December 2024).

## 4. Discussion

Retinal dysplasia is the term used to denote disorderly proliferation and imperfect differentiation of the developing retina, which can be further subdivided into focal and multifocal. Inherited forms of retinal disease are among the most well-characterised genetically in dogs [1]. The retinal dysplasia is a heterogeneous group of conditions that present with a range of severity of lesions, from those that do not affect vision [2] to those that result in blindness. Although in most cases retinal dysplasia lesions is observed in an otherwise normal eye, it may sometimes accompany other ocular malformations including persistent pupillary membrane or persistent hyperplastic primary vitreous. In some individuals affected with RD multiple ocular defects are present. Multifocal retinal dysplasia is described on ophthalmoscopic examination as retinal “folds”; grey or green linear or curvilinear streaks (or sometimes V- or Y-shaped streaks), areas of reduced tapetal reflectivity. Folds may be observed anywhere in the tapetal region, but most frequently occur in the central fundus, around the dorsal retinal vessels. The population of the Czechoslovakian Wolfdog has yet to be the subject of a purposeful study to determine the prevalence of individual eye diseases, including retinal dysplasia (RD). The American College of Veterinary Ophthalmologists [5] reports that detecting retinal folds in Czechoslovakian Wolfdog was limited to one out of thirty-five individuals examined from 1993–2018. The genetic background of this breed may provide a predisposition to ocular diseases characteristic of the German Shepherd. In the course of our study, we examined 117 Czechoslovakian Wolfdogs. In none of the dogs included in the study vision impairment was diagnosed. In all cases both neuro-ophthlamic and behavioural vision test were normal. In all dogs included in the study, no other ocular lesions have been observed. In adult Czechoslovakian Wolfdogs the proportion of retinal damage, considered as retinal folds was 3.59%. Described changes were observed in both tapetal and nontapetal fundus. In our study, three puppies in which ocular folds were diagnosed underwent a second ocular examination in the age of 6 months. During follow up, no ocular changes were found, changes may resolve with time as the retina and sclera grow and develop. In some breeds, including the Rough Collie and Shetland Sheepdogs, retinal folds may become less apparent or disappear as the animals mature [16]. According to the authors knowledge, it is the first report of retinal folds that may resolve in the Czechoslovakian Wolfdog.

The results of our genome-wide case–control association study revealed one SNP on CFA37 (BICF2G630130992, *p* = 1.29 × 10^−6^) localised within the first intron of the *CYP27A1* gene that was significantly associated with retinal dysplasia in Czechoslovakian Wolfdog. The cytochrome P450 27A1 gene (*CYP27A1*) encodes a mitochondrial enzyme sterol 27-hydrolase that is expressed in a wide variety of tissues and cell types including the retina [17] and plays an important role in the metabolism of cholesterol and cholesterol-related compounds [18]. Although it plays an important role in the metabolism of cholesterol and cholesterol-related compounds, its mutations have been linked to the occurrence in humans of the autosomal recessive disease cerebrotendinous xanthomatosis [19]. This disease is also characterised by the onset of clinical symptoms which can cause ocular manifestations such as cataracts and retinal degeneration. The knockout of the *CYP27A1* gene in a mouse model [20] led to dysregulation of retinal cholesterol homeostasis, including unexpected upregulation of retinal cholesterol biosynthesis and caused abnormal vascularisation in the retina. This finding may justify further investigation into this breed’s genetic basis of retinal dysplasias.

The other candidate gene associated with eye disease in the dog, localised in the region of the BICF2G630130992 is the *NHEJ1* gene. The position is ∼254,000 base pairs downstream of the SNP and encodes the non-homologous end-joining factor 1. The *NHEJ1* gene is a DNA repair factor essential for the non-homologous end-joining (NHEJ) pathway, which preferentially mediates the repair of DSBs. Failure to repair DSBs results in genetic instability, developmental delay, and immunodeficiency [21]. A comprehensive study identified a mutation in the *NHEJ1* gene, comprising a 7799 bp deletion within the fourth intron [22]. This mutation has been linked to the onset of clinical symptoms associated with CEA disease in certain breeds of sheepdogs, including the Collie, Border Collie, and Shetland Sheepdog. The Collie Eye Anomality (CEA) represents a complex trait, characterised by the disturbance of the chorioretinal and scleral development pattern. The primary aspect of the phenotype, designated as choroidal hypoplasia, presents as a localised defect of choroidal development in the temporal quadrant of the ocular fundus [22].

Another potential candidate gene in the region is the *CRYBA2* gene, which is situated approximately 173,000 base pairs downstream of the BICF2G630130992. The *CRYBA2* gene is a member of the crystallin gene family and is expressed in lens epithelial cells and fibre cells [23]. The crystallins are essential for the maintenance of lens transparency and functionality. The stability of the crystallin encoded by the mutant gene is compromised, resulting in lens opacity. Mammalian lens crystallins are classified into three main families: alpha, beta, and gamma. Among these, the beta crystallin family is the most heterogeneous [24]. Puk et al. [25] identified the first mutation in the *CRYBA2* gene in the mouse and proposed that the *CRYBA2* gene should be considered a strong candidate gene for age-related cataracts.

Two potential candidate genes, *CXCR1* and *CXCR2*, are located approximately 517,000 and 992,000 base pairs upstream of the BICF2G630130992 position, respectively. Both genes belong to the C-X-C motif chemokine receptor family. Goczalik et al. [26] monitored changes in an animal model of proliferative vitreoretinopathy in rabbit eyes and confirmed prominent expression of *CXCR1* and *CXCR2* genes, thereby suggesting additional physiological functions. The study by Garcia et al. [27] concluded that chronic CXCR2 deficiency in mice contributes to functional damage to the retina.

The pilot genome-wide case–control association study of 36 individuals from our national Czechoslovakian Wolfdog breed was conducted to optimise procedures for a more objective evaluation of phenotypic data obtained from clinical testing, to optimise individual steps in the genome-wide case–control association study procedure, and to determine the financial difficulty of a comprehensive analysis in a larger study. An initial correction of the data from the Illumina CanineHD 230K BeadChip provided us with information on 108,070 reliably determined genetic markers for the assessment of a particular breed. However, the results of our study are weighted with lower reliability due to the overall small number of individuals analysed and the small number of individuals in the case set. It is also not possible to generalise our results to the entire population of the Czechoslovakian Wolfdog breed. Apart from the negatives mentioned above, due to the small number of tested individuals, the use of a complex procedure of clinical examination of the ocular apparatus in combination with the inclusion of DNA information of a specific individual is a significant advance in the discovery of genetic predisposition.

In the investigation of genetic predispositions for an eye disease such as retinal dysplasia, it is essential to consider that although the clinical signs are identical across breeds, the genetic background of the disease is frequently distinct. It is therefore not appropriate to employ the routine use of molecular diagnostics of already documented causal mutations for this disease across breeds. The use of whole-genome association case–control studies will facilitate the specification of a region of the genome that can be subjected to more detailed analysis using large-scale sequence analyses with next-generation sequencing.

## 5. Conclusions

Retinal dysplasia is a common eye disease in some breeds of dogs. In contrast to the fact that the Czechoslovakian Wolfdog is a relatively healthy breed in terms of ocular disease, clinical examinations of 117 dogs revealed six individuals with multifocal retinal dysplasia. Through a genome-wide case–control association study performed on a selected group of 36 individuals, we identified the region of chromosome CFA37 that was potentially associated with retinal dysplasia in the Czechoslovakian Wolfdog breed. The localization of the SNP marker BICF2G630130992 in the intron of the *CYP27A1* gene allowed us to consider this gene as a potential candidate gene for retinal dysplasia in the breed. The region around the *CYP27A1* gene included other genes associated with ocular diseases in dogs or animal models such as rabbit, rat, and mouse. However, our results require further validation using next-generation sequencing approaches taking into account the region of interest chromosome CFA37 discovered in our study. This would allow us to more precisely characterize the genetic variations in that region and reveal their exact role in the development of retinal dysplasia in the Czechoslovakian Wolfdog.

## Figures and Tables

**Table 1:** Characteristics of samples representing a group of dogs clinically positive for multifocal form of retinal dysplasia.

Sample	Sex	Age	Number of Clinical Findings	Relative Value of the Damaged Area	Categorisation *
**1**	♂	2	13	0.014131804	**1.36**
**2**	♀	2	6	0.007484872	**0.75**
**3**	♂	2	62	0.010281335	**0.98**
**4**	♂	2	7	0.04961459	**2.84**
**5**	♀	4	4	0.009553546	**0.84**
**6**	♀	11	85	0.04862111	**2**

* Categorisation calculated using a regression model (R-square = 0.936).

## Data Availability

The original contributions presented in this study are included in the article. Further inquiries can be directed to the corresponding author.
